# Differentiated fatty acid allocation of *Daphnia magna* helped to maintain their population under food quality deterioration

**DOI:** 10.3389/fmicb.2025.1544005

**Published:** 2025-03-10

**Authors:** Sirui Wang, Zhengwen Liu, Xiaoqi Su, Xiaotong Jin, Hui Jin, Yaling Su, Jianjun Wang, Erik Jeppesen, Xiufeng Zhang, Yali Tang

**Affiliations:** ^1^Department of Ecology, Jinan University, Guangzhou, China; ^2^State Key Laboratory of Lake and Environment, Nanjing Institute of Geography and Limnology, Nanjing, China; ^3^Department of Ecoscience & WATEC, Aarhus University, Aarhus, Denmark; ^4^Sino-Danish Centre for Education and Research, Beijing, China; ^5^Limnology Laboratory, Department of Biological Sciences and Centre for Ecosystem Research and Implementation (EKOSAM), Middle East Technical University, Ankara, Türkiye; ^6^Institute of Marine Sciences, Middle East Technical University, Mersin, Türkiye; ^7^Institute for Ecological Research and Pollution Control of Plateau Lakes, School of Ecology and Environmental Sciences, Yunnan University, Kunming, China

**Keywords:** zooplankton, *Microcystis*, food quality, compound-specific stable isotope analysis, polyunsaturated fatty acids

## Abstract

Polyunsaturated fatty acids (PUFAs) are vital to the physiological functioning of crustacean zooplankton. However, cyanobacteria blooms frequently lead to PUFA deficiencies, which poses a substantial challenge to population fitness. Therefore, we hypothesize that *D. magna* adapt to PUFA-deficient conditions by prioritizing PUFA allocation to somatic growth, and then to offspring during reproduction to ensure population persistence. To test this hypothesis, we applied (compound-specific) ^13^C labeling to compare the turnover of total carbon and certain groups of fatty acids in *Daphnia magna* fed with *Scenedesmus bijuba* for 6 days and then switching to a diet of ^13^C labeled *Microcystis wesenbergii* for 6 days (with food quality deterioration) or to a diet of ^13^C-labeled *Scenedesmus* (without food quality deterioration), respectively. Fatty acid profiles of *D. magna* mothers and offspring were also analyzed to reveal their PUFA allocation strategies. Life table parameters from *D. magna*-feeding *Scenedesmus* switching to *Microcystis* were compared with *D. magna* fed with only *Scenedesmus* or *Microcystis* to reveal the effect of PUFA allocation on *D. magna* performance. Our results showed that with food quality deterioration, *D. magna* exhibited a significantly lower PUFA and carbon turnover and higher offspring: mother ratios in their PUFA contents. Despite this reduced reproduction, the *D. magna* switching diets showed no significant different intrinsic increasing rate of populations with those fed only *Scenedesmus.* Meanwhile, the *D. magna* switching diets performed significantly better than *D. magna* fed only *Microcystis*. These results suggest that differential fatty acid allocation of consumers may serve as an adaptive strategy for population maintenance in food quality deterioration and provide ecological implications with cyanobacterial bloom management and *Daphnia* reproductive plasticity, which needs further explorations.

## 1 Introduction

Consumers take up energetic and material resources as biochemicals from the environment, and after assimilation, biochemicals will enter either the structural pool to build biomass or the metabolic pool to produce energy (Brown et al., 2004). These biochemicals can be divided into two groups: essential and non-essential biochemicals (Kainz et al., 2009). Essential biochemicals are certain chemicals such as polyunsaturated fatty acids that are crucial for various physiological processes of consumers, such as membrane fluidity, the immune response, and the reproduction process (Valentine and Valentine, 2004; Stanley, 2000; Heckmann et al., 2008), but which cannot be synthesized *de novo* by them (Kainz et al., 2004; Twining et al., 2021). Lack of essential biochemicals causes food quality constraints and negatively affects the performance of consumers (Müller-Navarra et al., 2000; Ruess and Müller-Navarra, 2019; Twining et al., 2021; Tang et al., 2023). Hence, essential biochemicals should be contained in the diet in sufficient amounts to ensure consumer fitness, while consumers can flexibly metabolize and synthesize non-essential biochemicals, allowing support through various alternative forms (Kainz et al., 2009).

Food constraints are commonly seen in nature. For example, cyanobacteria may be stimulated by eutrophication, global warming, or heat waves (Persson et al., 2007; Paerl and Huisman, 2008; Urrutia-Cordero et al., 2020). In natural lakes, cyanobacteria dominance also reduces the food quality of seston because cyanobacteria are deficient in the long-chained polyunsaturated fatty acids (PUFAs), vital for regulating cell function in animals, hence reduce the fitness of zooplankton (Müller-Navarra et al., 2000; Taipale et al., 2022; Calderini et al., 2023; Kim et al., 2024). In response, zooplankton may adopt adaptive strategies to food constraints to maintain fitness. For example, a diet with a high carbon: phosphorus ratio may increase the ingestion rate of *D. magna* leading to higher phosphorus uptake (Darchambeau, 2005). At the same time, the *D. magna* will increase their respiration and egestion rates to get rid of excess carbon (Burian et al., 2020). In nature, food substrates are not present as individual elements but as biochemical compounds, and consumers ingest biochemicals to satisfy their elemental needs. The content of essential biochemicals influences the food quality and outweighs the direct effects of mineral P limitation (Tang and Dam, 1999; Park et al., 2002; Becker and Boersma, 2005; Thomas et al., 2022). Therefore, regulation of the allocation of essential biochemicals is needed to maintain consumer fitness, but this is a topic that have attracted less interest among researchers than those pertaining to elemental phosphorus (Wacker and Martin-Creuzburg, 2007; Lukas and Wacker, 2014).

Essential biochemicals must be sufficiently supplied in diets and maintained in certain amounts in the structural pool for individual fitness due to their crucial role in key physiological processes (Kainz et al., 2009; Twining et al., 2021). Previous studies have shown that the essential PUFA content in zooplankton is significantly higher than in seston (Kainz et al., 2004). A ^13^C-labeled experiment showed a switch by *Daphnia* from a high quality diet (i.e., *Cryptomonas*) to a moderate quality diet (*Scenedesmus*), retained PUFA from their original diet source even after 14 days (Taipale et al., 2011). More efficient trophic retention of PUFA biomolecules by zooplankton was found in a eutrophic than in a mesotrophic lake (Taipale et al., 2022).

Except for somatic growth, reproduction also involves resource allocation strategies. PUFAs and other essential lipids play critical roles in hormonal regulation, including the synthesis of eicosanoids and juvenile hormones, which are involved in triggering reproductive mode changes (Bhathena, 2000; Abrusán et al., 2007; Kainz et al., 2009). As the content of PUFA provided from mother *Daphnia* would affect the growth of their offspring (Sperfeld and Wacker, 2015), reproduction has been found to be a major drain of fatty acids, especially essential fatty acids from female *D. magna* (Becker and Boersma, 2005). Certain PUFA contents were more homeostatic in eggs than in somatic tissues when daphnids were exposed to food quality constraints in a study by Wacker and Martin-Creuzburg (2007).

Consumers such as zooplankton may exhibit differential fatty acid allocation during somatic growth and reproduction to cope with food quality deterioration in the environment. To increase our understanding of fatty acid allocation strategies of consumers, we designed life table experiments to compare the growth and reproduction of *D. magna* exposed to three dietary treatments: a 6-day *Scenedesmus* diet followed by a 15-day *Microcystis* diet and an unchanged diet of exclusively *Scenedesmus* or *Microcystis*. Fatty acid profile analysis was combined with compound specific carbon stable isotope labeling to examine the PUFA retention in the *D. magna* fed with the different diets. Our study provides new insight into the survival mechanisms of lake primary consumers during periodically occurring food quality deterioration events, e.g., cyanobacteria blooms.

## 2 Materials and methods

### 2.1 Materials

To focus on the effect of food quality, the non-toxic cyanobacteria *Microcystis wesenbergii* (FACHB-1339; purchased from Institute of Hydrobiology, Chinese Academy of Sciences, China) and *Scenedesmus bijuba* (obtained from Algal Culture Collection at Jinan University, China) were cultivated in BG11 medium (Stanier et al., 1971) at 20 ± 1°C under a 12 L:12D h light regime. The stock culture of *D. magna magna* (provided by Jinan University, China) was kept at 20 ± 1°C under a 12 L:12D h light regime in M4 medium together with the green algae *Scenedesmus*.

To label *Scenedesmus* and *Microcystis*, 35 mg NaH^13^CO_3_ (98 atom % ^13^C) (ISOTEC, USA) were added to the BG11 growth medium in a logarithmic growth phase of 48 h. δ^13^C of *Scenedesmus* and *Microcystis* increased from −20.09‰ and − 19.8‰ to 3767.9‰ and 3692.5‰, respectively.

To better quantify the food supply in the experiments, algae cells in the BG11 growth medium were collected, freeze-dried, and ground into powdery particles that were stored in a fridge and used as food in the subsequent experiments.

### 2.2 Experimental design

#### 2.2.1 ^13^C-labling experiments

As the PUFA content of the *D. magna* in the *Microcystis* diet treatments was fairly low and no reproduction occurred, the ^13^C labeling experiment was omitted. For the other diet treatments, the experiments were conducted in 250 mL glass beakers filled with 200 mL M4 medium and 2 mg C L^−1^ powdery phytoplankton particles, with 36 replicates with ten *D. magna* per beaker in each treatment. During the switch to ^13^C-labeled diets, the *D. magna* were sampled randomly on a daily basis from 3 of the 36 replicates for δ^13^C analyses, while the remaining individuals were transferred to new beakers with freshly prepared food; offspring were removed daily. In order to get sufficient samples for compound specific stable isotope analyses, we pooled the remaining *D. magna* samples.

#### 2.2.2 *Daphnia magna* fatty acid allocation experiment

Due to the insufficient biomass of *D. magna* offspring obtained in life table experiment, we carried out a batch experiment to obtain a higher biomass of maternal *D. magna* and offspring for fatty acids analyses. We used the same three diet treatments as in the life table experiments at a food concentration of 2 mg C L^−1^ for 21 days. One hundred *D. magna* offspring (< 24 h of age) were placed in a 4-L water tank. The medium was changed, and the *D. magna* were fed every day. The offspring were harvested every day and the maternal *D. magna* at the end of the experiment and stored at -20°C; all samples were freeze-dried for fatty acid analyses.

#### 2.2.3 Life table experiments

*D. magna* juveniles (< 24 h of age) were used for the life table experiment. All *D. magna* were starved for three hours in distilled water to empty their intestines, after which they were exposed to three different diet treatments (Table 1). The experiment was conducted in 50 mL glass beakers filled with 40 mL M4 medium and 2 mg C L^−1^ powdery phytoplankton particles. The experiment lasted 21 days. Each treatment had four replicates with five *D. magna* individuals per beaker. The animals were transferred into new beakers with freshly prepared food every day. The offspring produced in each beaker was counted, removed, and collected on a daily basis, and the length of *D. magna* was measured under a Nikon microscope every day. At the end of our experiments, all maternal *D. magna* were harvested, freeze-dried, and weighed on a micrometer balance (ME5, Sartorius, Germany) to obtain the biomass of *D. magna* by applying the equation between body length and body weight observed in our lab.

**Table 1:** Dietary treatments in carbon incorporation experiments and life table experiments.

	Carbon incorporation experiments	Life table experiments
Treatment 1	6-day unlabeled *Scenedesmus* diet + 12-day labeled *Scenedesmus* diet	21-day Scenedesmus diet
Treatment 2	6-day unlabeled *Scenedesmus* diet + 12-day labeled *Microcystis* diet	6-day Scenedesmus diet + 15-day *Microcystis* diet

### 2.3 Data analysis

#### 2.3.1 Carbon isotope analysis

Carbon isotope analysis of *D. magna* samples was conducted using a stable isotope ratio mass spectrometer (DELTA V Advantage, Thermo Scientific, USA) at the Analytical Testing Center at Jinan University with an analytical precision of 0.06‰. The stable isotope analysis for specific fatty acids of *D. magna* was conducted at Key Laboratory of Global Change and Marine-Atmospheric Chemistry, State Oceanic Administration, China, using gas chromatography-combustion-isotopic ratio mass spectrometry (GC-c-IRMS, Thermo Finnigan, USA) with an analytical precision of 0.3‰. The δ^13^C values of all specific compounds of saturated fatty acids (SAFA), monounsaturated fatty acids (MUFA), and polyunsaturated fatty acids (PUFA) were averaged, corresponding to the δ^13^C value of SAFA, MUFA, and PUFA. The detailed fatty acids grouped to saturated fatty acids (SAFA), monounsaturated fatty acids (MUFA), and polyunsaturated fatty acids (PUFA) were listed in Supplementary Tables 1, 2.

Carbon and fatty acid turnover were used to measure the proportion of *D. magna* carbon and fatty acids changed to the ^13^C enriched ones, with new measurements being made after the diet switch. The calculation was conducted by Simmr in the R package.

#### 2.3.2 Fatty acids, glucose, protein, and elemental analyses

Lipids from freeze-dried, homogenized *Scenedesmus*, *Microcystis* (3–5 mg), and *D. magna* (2–3 mg) samples were extracted according to the method described in a previous study by Bligh and Dyer (1959) and measured using gas chromatography–mass spectrometry (TRACE GC–MS, Thermo Finnigan, USA) at Analytical and Testing Center at Jinan University. The lipids were identified by retention times and mass spectra. Fatty acids were quantified with Supelco 37 Component FAME mix. Identified fatty acids absent from the Supelco 37 Component FAME mix were quantified by comparison with an internal standard (C12:0 methyl esters).

Glucose concentrations in *Scenedesmus* and *Microcystis* were determined by the phenol-concentrated sulfuric acids method (Dubois et al., 1956). Protein concentrations of *Scenedesmus* and *Microcystis* were measured using BCA test kit (Nanjing Jiancheng Bioengineering Research Institute, China). Besides, to calculate the C:P ratio, the C and P proportions of *Scenedesmus* and *Microcystis* (%) were determined at Analytical and Testing Center at Jinan University using an elemental analyzer (Vario EL CUBE, Elementar, Germany) and Inductively Coupled Plasma Atomic Emission Spectra (iCAP ICP-AES, Thermo Scientific, USA).

#### 2.3.3 Calculations of life table parameters

The weight of *D. magna* was calculated relative to body length using the following equation:


$$W=0.0096\times L^{2.647}$$

where W and L are the dry weight (mg) and body length (mm) of *D. magna*, respectively.

Somatic growth rates of *D. magna* (day^−1^) were calculated as the increase of dry weight during the experiments according to the following equation (Park and Goldman, 2006):


$$G=\left(\ln W_{T}-\ln W_{T_{0}}\right)/\left(T-T_{0}\right)$$

where G represents the growth rates of *D. magna*, $W_{T_{0}}$ is dry weight of *D. magna* at the beginning of the experiment, $W_{T}$ is dry weight of *D. magna* at time T (day), and $\left(T-T_{0}\right)$ is the duration of the experiments in days.

The intrinsic rates of increase r (d^−1^) in the *D. magna* population were calculated using Euler’s formula:


$$1=\sum_{x=0}^{n}e^{-rx}\times l_{x}\times m_{x}$$

where x is the age or time interval (day), $l_{x}$ is the proportion of individuals surviving to age x, and $m_{x}$ represents the number of offspring produced per surviving female at age x.

#### 2.3.4 Statistical analyses

One-way analysis of variance (one-way ANOVA) was performed to compare the food quality (fatty acid, protein, and carbohydrate concentrations) of *Scenedesmus* and *Microcystis*, the compound specific stable isotope of *D. magna*, the *D. magna* content of fatty acids, and the carbon turnover in *D. magna* over time. We also used one-way ANOVA to compare the somatic growth rates, reproductive output, survival rate, and population intrinsic growth rate of *D. magna* in the different diet treatments. Correlation between PUFA content and *D. magna* reproduction was analyzed by linear regression analysis. All analyses were conducted in SPSS 18.0 software (NY, USA). All raw data and standardized data met the assumption of Levene’s test of homogeneity of variance, and treatment effects were tested using Duncan *post hoc* tests.

## 3 Results

### 3.1 Biochemical proxies of *Scenedesmus* and *Microcystis*

**Figure 1:**
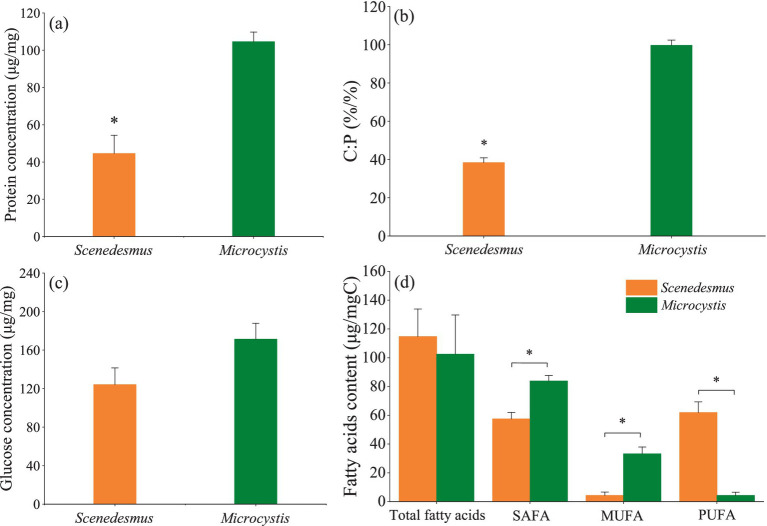
Food quality of *Scenedesmus* and *Microcystis* showing **(A)** protein concentration, **(B)** C:P ratio, **(C)** glucose concentration, and **(D)** total fatty acid, saturated fatty acid (SAFA), monosaturated fatty acid (MUFA) and polysaturated fatty acid (PUFA) concentrations of *Scenedesmus* and *Microcystis*. * indicates significant differences (*p <* 0.05).

The protein concentration and C:P mass ratio were significantly higher for *Microcystis* than for *Scenedesmus* (one-way ANOVA, *F* = 176.5, *p* < 0.001, Figure 1A; *F* = 538.1, *p* < 0.001, Figure 1B), while no significant difference was found for the glucose concentration and total fatty acids content between the two genera (one-way ANOVA, *F* = 1.89, *p* > 0.05, Figure 1C; *F* = 0.27, *p* > 0.05, Figure 1D). It should be noted that *Scenedesmus* had a much higher PUFA content (one-way ANOVA, *F* = 161.8, *p* < 0.001, Figure 1D) than *Microcystis*. Detailed fatty acid profiles of the two algal genera are presented in Supplementary Table 1.

### 3.2 Turnover of carbon and certain groups of fatty acid in *Daphnia magna*

**Figure 2:**
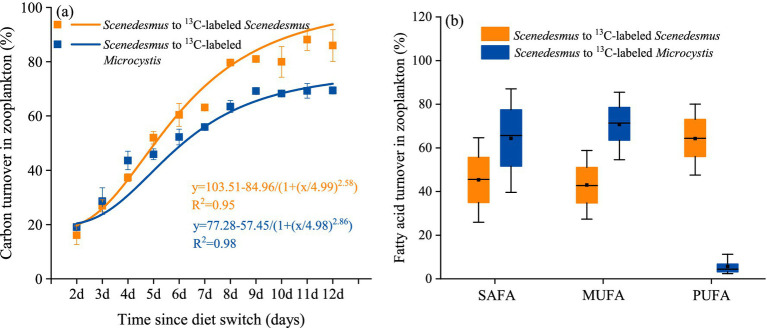
Carbon labeling results showing the turnover of **(A)** carbon in *D. magna* and **(B)** certain fatty acid group in *D. magna* in the pure *Scenedesmus* treatment and in the switch diet treatment.

After the diet switch, the carbon incorporation of phytoplankton by *D. magna* increased significantly with time (one-way ANOVA followed by Duncan’s test, *F* = 370.948, *p* < 0.001, Figure 2A). The contribution of *Microcystis* carbon to *D. magna* growth finally reached 69.4%. With the *Scenedesmus* diet (6-day unlabeled *Scenedesmus* switching to labeled *Scenedesmus*), the contribution of labeled *Scenedesmus* carbon also increased significantly with time (one-way ANOVA followed by Duncan’s test, *F* = 227.6, *p* < 0.001, Figure 2A). At the end of the experiment, the contribution of *Scenedesmus* carbon to *D. magna* had reached 89.3%. The turnovers of SAFA, MUFA, and PUFA were all >40% in the *D. magna* fed with *Scenedesmus* experiment. The turnovers of SAFA and MUFA in the diet shift experiment were > 60%; however, turn over of PUFA was <5%.

### 3.3 Polyunsaturated fatty acids allocation in *Daphnia magna*

**Figure 3:**
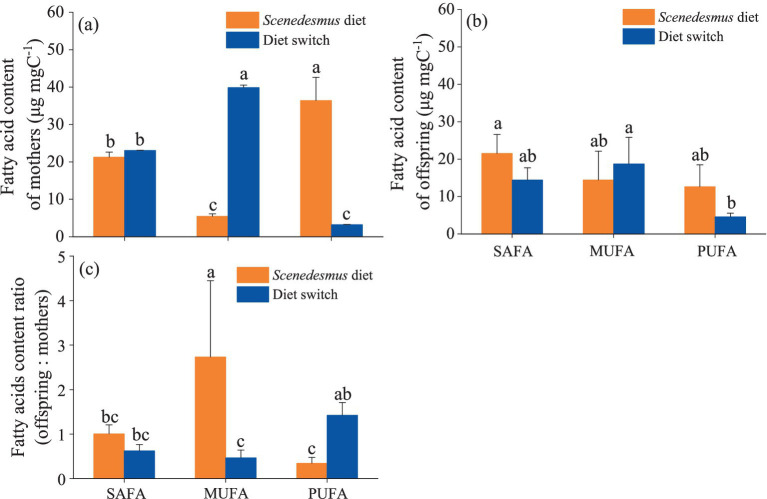
Polyunsaturated fatty acids (PUFA) content of *D. magna* showing saturated fatty acid (SAFA), mono-saturated fatty acid (MUFA), and poly-unsaturated fatty acid (PUFA) contents in *D. magna* **(A)** mothers and **(B)** offspring (* indicates absence of offspring), and **(C)** MUFA, SAFA, and PUFA allocation differences between *D. magna* mothers and offspring. Treatments sharing the same letter indicate no significant differences (*p* > 0.05).

Detailed fatty acid profiles of *D. magna* (mothers and offspring) in the different diet treatments are presented in Supplementary Table 2. The PUFA content of *D. magna* mothers fed with a switching diet was significantly higher than that in *D. magna* reared on a constant *Microcystis* diet and significantly lower than for the *D. magna* fed with only *Scenedesmus* (one-way ANOVA followed by Duncan’s test, *F* = 87.6, *p* < 0.001). All fatty acid groups were more homeostatic in offspring than in mothers, with no significant differences in the SAFA, MUFA, and PUFA content of the offspring (one-way ANOVA followed by Duncan’s test, all *p* > 0.05) between the two treatments (Figure 3A). The PUFA_offspring_:PUFA_mother_ ratio was significantly higher in the switching diet treatment than in the constant *Scenedesmus* diet treatment (one-way ANOVA followed by Duncan’s test, *F* = 8.12, *p* = 0.001; Figure 3B).

### 3.4 *Daphnia magna* growth experiments

**Figure 4:**
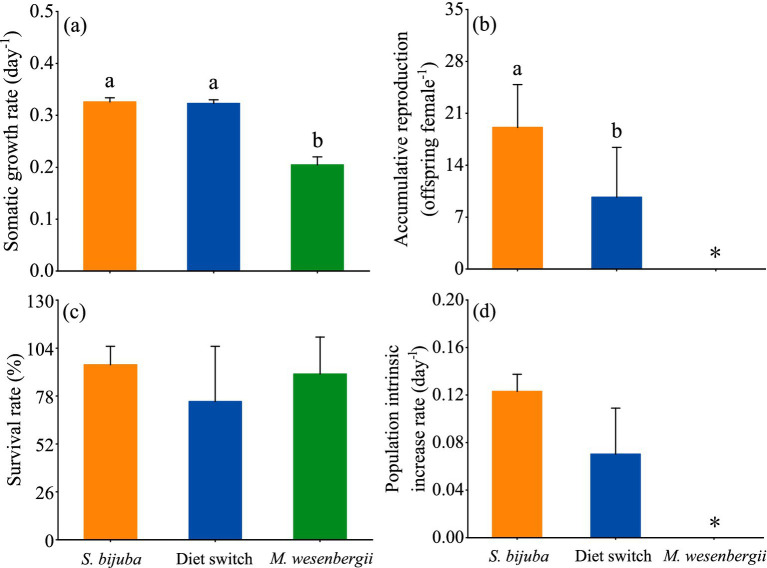
Life history of *D. magna* showing **(A)** somatic growth rates before reproducing, **(B)** accumulative reproduction during the whole experimental period (* indicating absence of offspring in the treatment), **(C)** survival rate at the end of the experiments, and **(D)** population intrinsic increase rate (* indicating that data could not be calculated by Euler’s formula due to 0 reproduction). Treatments sharing the same letter indicate no significant differences (*p* > 0.05).

Before reproducing, the growth rates of *D. magna* fed exclusively with *Scenedesmus* and switching diets were significantly higher than the growth rate of *D. magna* fed exclusively with *Microcystis* diet, whereas the growth rates of *D. magna* showed no statistical differences between the *Scenedesmus* treatment and the switched diet treatment (one-way ANOVA followed by Duncan’s test, *F* = 405.9, *p* < 0.001; Figure 4A). Significant differences in the reproductive output between the *D. magna* in the constant *Scenedesmus* treatment and the switched diet treatment were also observed (one-way ANOVA followed by Duncan, *F* = 11.280, *p* < 0.01; Figure 4B). *D. magna* fed constantly with *Scenedesmus* produced, on average, 19.1 offspring, being significantly higher than the *D. magna* fed with the switched diet (on average 9.7). No offspring occurred in the constant *Microcystis* diet treatment during the experimental period (Figure 4B). There were no significant differences in the population intrinsic increase rate of *D. magna* between the constant *Scenedesmus* diet treatment and the switched diet treatment (one-way ANOVA, *F* = 6.29, *p* > 0.05; Figure 4D).

## 4 Discussion

Based on the results of the 13C-labeling, fatty acid allocation detection, and life table experiments, we observed low PUFA turnover and increased PUFA allocation to offspring, supporting the hypothesis that *D. magna* exhibit adaptive strategies to maintain population fitness under food quality constraints.

At food quality constraint, consumers may preserve carbon from their initial unconstrained diet, which we observed in the carbon turnover in our feeding experiments after the diet switch. In our *Scenedesmus* diet shifting to labeled *Scenedesmus* dietary treatment, the carbon turnover was nearly 100% indicating that *D. magna* consumed biochemicals from their initial diet for energy, while storing the biochemicals from the labeled diet. However, 60% carbon turnover in the *Scenedesmus* shifting to *Microcystis* dietary treatment indicates that 40% carbon from the unlabeled *Scenedesmus* diet was retained in body biomass of *D. magna*. The organic carbon (i.e., biochemicals) including carbohydrates, fatty acids and proteins could alternatively be allocated to excretion for survival, energy acquisition for respiration, or biomass for maintenance of some crucial physiological process, e.g., membrane fluidity after assimilation by consumers (Lukas and Wacker, 2014; Burian et al., 2020). Consumers tend to allocate high quality food to their biomass (Kühmayer et al., 2020; Tang et al., 2021). The retention of good quality carbon can be due to physiological constraints of disability of metabolizing essential biochemicals or elements, or be an active adaptive mechanism of increased metabolizing non-essential biochemicals by consumers. *Scenedesmus* are better quality food than *Microcystis* (Gulati and DeMott, 1997), with significantly higher protein content, C: P ratio and more importantly, PUFA content.

PUFA are important components of the cell membrane that influence fluidity, affecting cellular communication by modulating membrane-bound enzymes and receptors (Brett and Müller-Navarra, 1997; Heckmann et al., 2008; Twining et al., 2021). For the physiological constraints to maintain cellular integrity, PUFA should be kept in consumers’ biomass in a certain amount (Kainz et al., 2004). This explains our results of low PUFA turnover when switching diet from *S. bijuba* to *M. wesenbergii.* Low PUFA turnover indicates that PUFA of *D. magna* was obtained from the initial *S. bijuba* diet. Besides, we estimated the PUFA retention by comparing the PUFA concentration of *D. magna* fed the switch diet to those fed only *M. wesenbergii* diet. Taking together, we confirmed that PUFA originated from former *S. bijuba* was retained. This also led to the speculations that PUFA contributed to the unchanged part of carbon. To obtain enough samples for stable isotopic analysis for different fatty acid groups, all *D. magna* from various days after diet switch were mixed, resulting in a lower fatty acid turnover compared to the carbon turnover. Still, the lowest fatty acid turnover of PUFA among all the fatty acid groups occurred from the *D. magna* fed with *Scenedesmus* shifting to *Microcystis.* At the constant *Scenedesmus* diet, *D. magna* had a higher average turnover of PUFA than of SAFA and MUFA. This could be due to the accumulation of the latter PUFA supply as described before (Taipale et al., 2011), or the metabolization of the former assimilated PUFA, or both situations. Our study revealed significantly elevated PUFA concentrations of *D. magna* fed constant *Scenedesmus* comparing to those in the switch dietary treatment, evidencing the accumulation of the latter dietary supplied PUFA. The accumulation of PUFA can be explained by their role as precursors of eicosanoids, including prostaglandins, leukotrienes, and thromboxanes, which exhibit hormone-like activities and regulate immune responses and the reproduction process such as the physiology of leuteolysis and ovulation (Bhathena, 2000; Stanley, 2006). Besides, PUFAs has been documented to attenuate the production of reactive oxygen species (ROS) and elevate glutathione peroxidase activity, protecting cells from stress-induced damage (Haimeur et al., 2012).

PUFA is not only allocated for somatic growth but also for reproduction. In our study, we observed more homeostatic PUFA contents in offspring than in mothers, corresponding to a previous study (Wacker and Martin-Creuzburg, 2007). Moreover, our results showed highly increased offspring:mother PUFA ratios under PUFA constraints, indicating that PUFA allocation to offspring is a first priority under such circumstance. Generally, except their genotype and present environment, organisms may also be profoundly affected by the environment that has been experienced by previous generations, which is called non-genetic trans-generational process (Mousseau and Fox, 1998; Zhou and Declerck, 2020). When nutritional conditions deteriorate, consumers can change the allocation patterns of energy resources between mothers and offspring, a process known as anticipatory maternal effects (Marshall and Uller, 2007; Wacker and Martin-Creuzburg, 2007). Sperfeld and Wacker (2015) showed that the mass-specific growth responses of offspring to their diets were affected by the maternal diet regime and speculated that this reflects varying maternal PUFA provisioning and that maternal provisioning of PUFA helps to prevent growth limitation of offspring until maturity is reached (Sperfeld and Wacker, 2015). The privilege of offspring PUFA allocation under food quality constraints might be one reason for anticipatory maternal effects, providing opportunities for the population to survive longer under nutritional constraints.

The life table experiments revealed that the adjustments to fatty acids benefited the somatic growth, reproduction, and population growth of *D. magna* experiencing food quality constraints. *Microcystis* alone did not support the reproduction of *D. magna*, resulting in extinction of the population, as previously described (Luo et al., 2015). Ephippia might be produced in this treatment, but no larvae was hatched during the experiment. Nevertheless, although an initial 6-day diet of *Scenedesmus* did not supply efficient PUFA, the PUFA present were strictly budgeted. As a result, a significantly higher reproduction rate of *D. magna* occurred in the *Scenedesmus*-alone than in the *Microcystis-*alone treatment. We also observed a non-significant population intrinsic increase rate in the *Scenedesmus* shifting *to Microcystis* treatment compared to the *Scenedesmus-*alone treatment, which could be that sample size is sufficient to detect subtle differences. But more importantly, comparing to *D. magna* fed *Microcystis* alone, the switch diet actually show simultaneously the PUFA retention in body biomass and offspring, as well as significantly improved population increase.

Cyanobacteria can have toxicity effect to zooplankton, but that negative effect can be species−/clone-specific (Ger et al., 2014). So research focused on food quality perspective was also a hot topic (Ravet et al., 2010; Taipale et al., 2011; Taipale et al., 2022). In field experiments, the PUFA contents of *D. magna* has been shown to be higher than those of their food items, indicating retention of PUFA (Ravet et al., 2010; Taipale et al., 2022). In lab experiments, *D. magna* exhibited long-term retention of eicosapentaenoic (EPA; 20:5 ω3) and arachidonic acid (ARA; 20:4 ω6) when switching from a *Cryptomonas* to a *Scenedesmus* diet (Taipale et al., 2011). Our study focused on quick and sharp food quality changes induced by cyanobacteria blooms and we found that differentiated fatty acid allocation occurs during both somatic growth and reproduction, emphasize the role of fatty acid allocation strategies for zooplankton population dynamics. These findings provide ecological implications with cyanobacterial bloom management and *Daphnia* reproductive plasticity, which needs further explorations.

## Data Availability

The original contributions presented in the study are included in the article/Supplementary material, further inquiries can be directed to the corresponding authors.

## References

[ref1] Abrusán G. Fink P. Lampert W. (2007). Biochemical limitation of resting egg production in Daphnia. Limnol. Oceanogr. 52, 1724–1728. doi: 10.4319/lo.2007.52.4.1724

[ref2] Becker C. Boersma M. (2005). Differential effects of phosphorus and fatty acids on *Daphnia magna* growth and reproduction. Limnol. Oceanogr. 50, 388–397. doi: 10.4319/lo.2005.50.1.0388

[ref3] Bhathena S. J. (2000). Relationship between fatty acids and the endocrine system. Biofactors 13, 35–39. doi: 10.1002/biof.5520130107 11237196

[ref4] Bligh E. G. Dyer W. J. (1959). A rapid method of total lipid extraction and purification. Can. J. Biochem. Physiol. 37, 911–917. doi: 10.1139/y59-099 13671378

[ref5] Brett M. Müller-Navarra D. (1997). The role of highly unsaturated fatty acids in aquatic foodweb processes. Freshw. Biol. 38, 483–499. doi: 10.1046/j.1365-2427.1997.00220.x

[ref6] Brown J. H. Gillooly J. F. Allen A. P. Savage V. M. West G. B. (2004). Toward a metabolic theory of ecology. Ecology 85, 1771–1789. doi: 10.1890/03-9000

[ref7] Burian A. Nielsen J. M. Winder M. (2020). Food quantity–quality interactions and their impact on consumer behavior and trophic transfer. Ecol. Monogr. 90:e01395. doi: 10.1002/ecm.1395

[ref8] Calderini M. L. Kahilainen K. K. Estlander S. Peltomaa E. Piro A. J. Rigaud C.. (2023). Eutrophication effect on production and transfer of omega-3 fatty acids in boreal lake food webs. Sci. Total Environ. 903:166674. doi: 10.1016/j.scitotenv.2023.166674, PMID: 37647960

[ref9] Darchambeau F. (2005). Filtration and digestion responses of an elementally homeostatic consumer to changes in food quality: a predictive model. Oikos 111, 322–336. doi: 10.1111/j.0030-1299.2005.13497.x

[ref10] Dubois M. Gilles K. A. Hamilton J. K. Rebers P. T. Smith F. (1956). Colorimetric method for determination of sugars and related substances. Anal. Chem. 28, 350–356. doi: 10.1021/AC60111A017

[ref11] Ger K. A. Hansson L. A. Lürling M. (2014). Understanding cyanobacteria-zooplankton interactions in a more eutrophic world. Freshw. Biol. 59, 1783–1798. doi: 10.1111/fwb.12393

[ref12] Gulati R. DeMott B. (1997). The role of food quality for zooplankton: remarks on the state-of-the-art, perspectives and priorities. Freshw. Biol. 38, 753–768. doi: 10.1046/j.1365-2427.1997.00275.x

[ref13] Haimeur A. Ulmann L. Mimouni V. Guéno F. Pineau-Vincent F. Meskini N.. (2012). The role of *Odontella aurita*, a marine diatom rich in EPA, as a dietary supplement in dyslipidemia, platelet function and oxidative stress in high-fat fed rats. Lipids Health Disease 11, 1–13. doi: 10.1186/1476-511X-11-147, PMID: 23110391 PMC3543224

[ref14] Heckmann L. Sibly R. M. Timmermans M. J. Callaghan A. (2008). Outlining eicosanoid biosynthesis in the crustacean Daphnia. Front. Zool. 5, 11–19. doi: 10.1186/1742-9994-5-11, PMID: 18625039 PMC2483973

[ref15] Kainz M. Arts M. T. Mazumder A. (2004). Essential fatty acids in the planktonic food web and their ecological role for higher trophic levels. Limnol. Oceanogr. 49, 1784–1793. doi: 10.4319/lo.2004.49.5.1784

[ref16] Kainz M. Brett M. T. Arts M. T. (2009). Lipids in aquatic ecosystems. 1st Edn. New York: Springer.

[ref17] Kim J. O. Dimitriou A. Forster I. Tseng M. (2024). Heatwave-mediated decreases in phytoplankton quality negatively affect zooplankton productivity. Funct. Ecol. 38, 778–791. doi: 10.1111/1365-2435.14530

[ref18] Kühmayer T. Guo F. Ebm N. Battin T. J. Brett M. T. Bunn S. E.. (2020). Preferential retention of algal carbon in benthic invertebrates: stable isotope and fatty acid evidence from an outdoor flume experiment. Freshw. Biol. 65, 1200–1209. doi: 10.1111/fwb.13492, PMID: 32612313 PMC7317824

[ref19] Lukas M. Wacker A. (2014). Daphnia’s dilemma: adjustment of carbon budgets in the face of food and cholesterol limitation. J. Exp. Biol. 217, 1079–1086. doi: 10.1242/jeb.094151, PMID: 24311814

[ref20] Luo X. Liu Z. Gulati R. D. (2015). Cyanobacterial carbon supports the growth and reproduction of Daphnia: an experimental study. Hydrobiologia 743, 211–220. doi: 10.1007/s10750-014-2038-7

[ref21] Marshall D. J. Uller T. (2007). When is a maternal effect adaptive? Oikos 116, 1957–1963. doi: 10.1111/j.2007.0030-1299.16203.x

[ref22] Mousseau T. A. Fox C. W. (1998). The adaptive significance of maternal effects. Trends Ecol. Evol. 13, 403–407. doi: 10.1016/S0169-5347(98)01472-4 21238360

[ref23] Müller-Navarra D. C. Brett M. T. Liston A. M. Goldman C. R. (2000). A highly unsaturated fatty acid predicts carbon transfer between primary producers and consumers. Nature 403, 74–77. doi: 10.1038/47469, PMID: 10638754

[ref24] Paerl H. W. Huisman J. (2008). Blooms like it hot. Science 320, 57–58. doi: 10.1126/science.1155398, PMID: 18388279

[ref25] Park S. Brett M. T. Müller-Navarra D. C. Goldman C. R. (2002). Essential fatty acid content and the phosphorus to carbon ratio in cultured algae as indicators of food quality for Daphnia. Freshw. Biol. 47, 1377–1390. doi: 10.1046/j.1365-2427.2002.00870.x

[ref26] Park S. K. Goldman C. R. (2006). Carbon assimilation and respiration of *Daphnia magna* with varying algal food quality. J. Ecol. Environ. 29, 433–438. doi: 10.5141/JEFB.2006.29.5.433

[ref27] Persson J. Brett M. T. Vrede T. Ravet J. L. (2007). Food quantity and quality regulation of trophic transfer between primary producers and a keystone grazer (Daphnia) in pelagic freshwater food webs. Oikos 116, 1152–1163. doi: 10.1111/j.0030-1299.2007.15639.x

[ref28] Ravet J. L. Brett M. T. Arhonditsis G. B. (2010). The effects of seston lipids on zooplankton fatty acids composition in Lake Washington, Washington, USA. Ecology 91, 180–190. doi: 10.1890/08-2037.1 20380207

[ref29] Ruess L. Müller-Navarra D. C. (2019). Essential biomolecules in food webs. Front. Ecol. Evol. 7:269. doi: 10.3389/fevo.2019.00269

[ref30] Sperfeld E. Wacker A. (2015). Maternal diet of *Daphnia magna* affects offspring growth responses to supplementation with particular polyunsaturated fatty acids. Hydrobiologia 755, 267–282. doi: 10.1007/s10750-015-2244-y

[ref31] Stanier R. Y. Kunisawa R. Mandel M. C. B. G. Cohen-Bazire G. (1971). Purification and properties of unicellular blue-green algae (order Chroococcales). Bacteriol. Rev. 35, 171–205. doi: 10.1128/br.35.2.171-205.1971, PMID: 4998365 PMC378380

[ref32] Stanley D. W. (2000). Eicosanoids in invertebrate signal transduction systems. 1st Edn. Princeton University Press: Princeton.

[ref33] Stanley D. (2006). Prostaglandins and other eicosanoids in insects: biological significance. Annu. Rev. Entomol. 51, 25–44. doi: 10.1146/annurev.ento.51.110104.151021, PMID: 16332202

[ref34] Taipale S. J. Kainz M. J. Brett M. T. (2011). Diet-switching experiments show rapid accumulation and preferential retention of highly unsaturated fatty acids in Daphnia. Oikos 120, 1674–1682. doi: 10.1111/j.1600-0706.2011.19415.x

[ref35] Taipale S. J. Ventelä A. M. Litmanen J. Anttila L. (2022). Poor nutritional quality of primary producers and zooplankton driven by eutrophication is mitigated at upper trophic levels. Ecol. Evol. 12:e8687. doi: 10.1002/ece3.8687, PMID: 35342549 PMC8928886

[ref36] Tang K. W. Dam H. G. (1999). Limitation of zooplankton production: beyond stoichiometry. Oikos 84, 537–542. doi: 10.2307/3546434

[ref37] Tang Y. Su L. Xu R. Wang S. Su Y. Liu Z.. (2023). Response of zooplankton to inputs of terrestrial dissolved organic matter: food quality constraints induced by microbes. Limnol. Oceanogr. 68, 709–722. doi: 10.1002/lno.12304

[ref38] Tang Y. Zhou D. Su L. Liu Z. Zhang X. Dumont H. J. (2021). *Vallisneria natans* detritus supports *Daphnia magna* somatic growth and reproduction under addition of periphyton. Aquat. Microb. Ecol. 55, 579–588. doi: 10.1007/s10452-021-09846-5

[ref39] Thomas P. K. Kunze C. Van de Waal D. B. Hillebrand H. Striebel M. (2022). Elemental and biochemical nutrient limitation of zooplankton: a meta-analysis. Ecol. Lett. 25, 2776–2792. doi: 10.1111/ele.14125, PMID: 36223425

[ref40] Twining C. W. Bernhardt J. R. Derry A. M. Hudson C. M. Ishikawa A. Kabeya N.. (2021). The evolutionary ecology of fatty-acid variation: implications for consumer adaptation and diversification. Ecol. Lett. 24, 1709–1731. doi: 10.1111/ele.13771, PMID: 34114320

[ref41] Urrutia-Cordero P. Zhang H. Chaguaceda F. Geng H. Hansson L. A. (2020). Climate warming and heat waves alter harmful cyanobacterial blooms along the benthic–pelagic interface. Ecology 101:e03025. doi: 10.1002/ecy.3025, PMID: 32083737

[ref42] Valentine R. C. Valentine D. L. (2004). Omega-3 fatty acids in cellular membranes: a unified concept. Prog. Lipid Res. 43, 383–402. doi: 10.1016/j.plipres.2004.05.004, PMID: 15458813

[ref43] Wacker A. Martin-Creuzburg D. (2007). Allocation of essential lipids in *Daphnia magna* during exposure to poor food quality. Funct. Ecol. 21, 738–747. doi: 10.1111/j.1365-2435.2007.01274.x

[ref44] Zhou L. Declerck S. A. (2020). Maternal effects in zooplankton consumers are not only mediated by direct but also by indirect effects of phosphorus limitation. Oikos 129, 766–774. doi: 10.1111/oik.06898

