# Supplementary material for: Differentiated fatty acid allocation of Daphnia magna helped to maintain their population under food quality deterioration
Source: Front Microbiol. 2025 Mar 10;16:1544005. doi: 10.3389/fmicb.2025.1544005 (PMC11931139; doi:10.3389/fmicb.2025.1544005)
Supplement: Supplementary file 1 [file Table_1.docx]

**Supplementary table 1.** Fatty acids composition and content (μg/mgC) of *Scenedesmus* and *Microcystis.*

| Fatty acids profile | *S. bijuba* | *M. wesenbergii* |
| --- | --- | --- |
|  | μg/mgC | μg/mgC |
| Saturated fatty acids | | |
| C14:0 | 2.05 ± 0.00 | 1.50 ± 0.00 |
| C15:0 | - | 0.26 ± 0.20 |
| C16:0 | 45.07 ± 13.07 | 56.05 ± 20.93 |
| C17:0 | 0.63 ± 0.32 | 0.14 ± 0.05 |
| C18:0 | - | 9.43 ± 9.26 |
| C19:0 | - | 0.34 ± 0.24 |
| C20:0 | 0.32 ± 0.13 | 0.11 ± 0.00 |
| C21:0 | 0.47 ± 0.00 | 4.21 ± 0.00 |
| C22:0 | 0.30 ± 0.11 | - |
| C23:0 | 0.29 ± 0.00 | - |
| C24:0 | 0.50 ± 0.13 | 0.18 ± 0.00 |
| Monounsaturated fatty acids | | |
| C16:1ω3 | - | 0.68 ± 0.00 |
| C16:1ω5 | - | 0.71 ± 0.00 |
| C16:1ω7 | 2.68 ± 0.00 | 18.44 ± 11.69 |
| C16:1ω9 | 0.56 ± 0.33 | 2.21 ± 2.11 |
| C16:1ω14 | - | 0.31 ± 0.00 |
| C17:1ω7 | - | 0.30 ± 0.13 |
| C18:1ω7 | - | 6.05 ± 3.26 |
| C18:1ω9 | 2.82 ± 0.68 | 6.25 ± 3.57 |
| C19:1ω6 | - | 0.19 ± 0.00 |
| C19:1ω9 | - | 0.35 ± 0.00 |
| Polyunsaturated fatty acids | | |
| C16:2ω6 | 2.95 ± 0.47^a^ | 0.67 ± 0.20^b^ |
| C16:3ω3 | 2.25 ± 0.00 | 1.40 ± 0.50 |
| C16:3ω6 | 2.06 ± 0.54 | 0.36 ± 0.00 |
| C17:2ω6 | 2.26 ± 0.00 | - |
| C18:2ω6 | 10.53 ± 2.08^a^ | 2.66 ± 0.98^b^ |
| C18:2ω8 | 0.16 ± 0.00 | - |
| C18:3ω3 | 41.4 ± 5.35^a^ | 0.16 ± 0.03^b^ |
| C20:4ω6 | 0.35 ± 0.13 | - |
| C20:4ω9 | 0.46 ± 0.00 | - |
| C20:5ω3 | 2.26 ± 2.11 | - |
| C22:4ω7 | 1.07 ± 0.60 | - |

Note: "-"means trace amount (<0.1) or not detected.
