# Supplementary material for: Differentiated fatty acid allocation of Daphnia magna helped to maintain their population under food quality deterioration
Source: Front Microbiol. 2025 Mar 10;16:1544005. doi: 10.3389/fmicb.2025.1544005 (PMC11931139; doi:10.3389/fmicb.2025.1544005)
Supplement: Supplementary file 2 [file Table_2.docx]

**Supplementary table 2**. Fatty acid composition and content (μg/mgC) in zooplankton

|  | Mothers in *Scenedesmus* | Mothers in switch diet | Mothers in *Microcystis* | Offspring in *Scenedesmus* | Offspring in switch diet |
| --- | --- | --- | --- | --- | --- |
| Saturated fatty acids | | | | | |
| C13:0 | 0.12 ± 0.03^b^ | 0.35 ± 0.01^a^ | 0.37 ± 0.08^a^ | 0.08 ± 0.03^b^ | 0.13 ± 0.10^b^ |
| C14:0 | 2.38 ± 0.14^a^ | 2.49 ± 0.00^a^ | 2.06 ± 0.58^a^ | 2.00 ± 1.07^a^ | 0.79 ± 0.37^b^ |
| C15:0 | 1.45 ± 0.10^b^ | 2.54 ± 0.00^a^ | 1.80 ± 0.33^b^ | 1.58 ± 0.61^b^ | 1.13 ± 0.32^b^ |
| C16:0 | 15.11 ± 1.28^a^ | 14.99 ± 0.01^a^ | 12.03 ± 1.53^ab^ | 14.51 ± 3.61^a^ | 9.91 ± 2.66^b^ |
| C17:0 | 0.56 ± 0.14^b^ | 0.84 ± 0.04^a^ | 0.62 ± 0.10^b^ | 0.74 ± 0.14^ab^ | 0.53 ± 0.11^b^ |
| C18:0 | 1.59 ± 0.15 | 1.87 ± 0.04 | 2.42 ± 0.49 | 2.55 ± 0.87 | 1.97 ± 0.47 |
| C19:0 | 0.08 ± 0.00 | - | - | 0.08 ± 0.03 | - |
| C20:0 | 0.05 ± 0.00 | - | 0.05 ± 0.00 | 0.07 ± 0.00 | - |
| Monounsaturated fatty acids | | | | | |
| C14:1ω3 | 0.22 ± 0.12 | - | 0.38 ± 0.00 | - | 0.58 ± 0.40 |
| C14:1ω5 | 0.24 ± 0.00 | 1.99 ± 0.00 | 1.30 ± 0.43 | 0.25 ± 0.00 |  |
| C15:1ω5 | - | 0.28 ± 0.00 | 0.24 ± 0.03 | - | 0.15 ± 0.06 |
| C15:1ω7 | - | 0.09 ± 0.00 | - | - | - |
| C16:1ω5 | 0.75 ± 0.18^a^ | 0.57 ± 0.00^ab^ | 0.62 ± 0.26^ab^ | 0.56 ± 0.28^ab^ | 0.16 ± 0.13^b^ |
| C16:1ω6 | 0.16 ± 0.00 | 0.06 ± 0.00 | 0.04 ± 0.00 | - | - |
| C16:1ω7 | 0.11 ± 0.00 | 24.81 ± 0.33 | 15.46 ± 2.34 | 0.42 ± 0.00 | 10.49 ± 5.91 |
| C16:1ω9 | 2.40 ± 0.20 | 1.23 ± 0.34 | 0.72 ± 0.04 | 2.60 ± 1.30 | 3.27 ± 3.92 |
| C17:1ω7 | 0.17 ± 0.00 | 0.56 ± 0.00 | 0.31 ± 0.05 | 0.09 ± 0.00 | - |
| C17:1ω8 | 0.12 ± 0.00 | - | 0.37 ± 0.00 | - | 0.25 ± 0.07 |
| C18:1ω7 | 0.14 ± 0.00 | - | 1.43 ± 1.80 | - | - |
| C18:1ω9 | 1.61 ± 0.35^b^ | 10.53 ± 0.57^a^ | 6.76 ± 2.29^ab^ | 10.78 ± 8.73^a^ | 7.54 ± 2.07^ab^ |
| C18:1ω12 | - | 0.07 ± 0.00 | - | - | - |
| C22:1ω9 | - | 0.14 ± 0.09 | 0.29 ± 0.00 | 0.24 ± 0.09 | 0.13 ± 0.00 |
| C22:1ω11 | 0.19 ± 0.09 | 0.20 ± 0.00 | 0.29 ± 0.09 | 0.18 ± 0.00 | 0.18 ± 0.01 |
| Polyunsaturated fatty acids | | | | | |
| C16:2ω6 | 0.67 ± 0.04 | - | - | 0.44 ± 0.35 | 0.09 ± 0.00 |
| C16:3ω3 | 0.34 ± 0.03 | - | - | - | - |
| C16:3ω6 |  | - | - | 0.3 ± 0.00 | - |
| C16:4ω3 | 2.90 ± 0.10 | - | - | 1.98 ± 1.67 | 0.10 ± 0.00 |
| C18:2ω6 | 4.84 ± 0.84^a^ | 0.51 ± 0.54^b^ | 0.42 ± 0.45^b^ | 5.06 ± 0.90^a^ | 0.66 ± 0.48^b^ |
| C18:2ω7 | 5.76 ± 0.00 | 0.86 ± 0.00 | 0.46 ± 0.00 | 2.58 ± 0.00 | 1.31 ± 0.02 |
| C18:3ω3 | 23.12 ± 4.59 | - | - | - | - |
| C18:3ω6 | 0.31 ± 0.03 | 0.13 ± 0.00 |  | 0.37 ± 0.10 |  |
| C18:4ω3 | 1.94 ± 0.19 | 0.07 ± 0.00 | 0.13 ± 0.03 | 1.03 ± 0.79 | 0.05 ± 0.00 |
| C20:3ω6 | - | 0.18 ± 0.00 | 0.10 ± 0.00 | 0.08 ± 0.03 | 0.04 ± 0.00 |
| C20:4ω3 | 0.23 ± 0.00 | - | - | - | - |
| C20:4ω6 | 1.25 ± 0.51^b^ | 1.85 ± 0.01^ab^ | 1.24 ± 0.21^b^ | 2.89 ± 1.01^a^ | 2.76 ± 1.22^a^ |
| C20:5ω3 | 0.75 ± 0.26^b^ | 0.21 ± 0.00^b^ | 0.14 ± 0.12^b^ | 1.66 ± 0.66^a^ | 0.43 ± 0.17^b^ |
| C22:6ω3 | - | 0.22 ± 0.00 | 0.16 ± 0.00 | - | - |
